# A Streamlined Treatment Algorithm for Allergic Rhinitis in the Arab Region: Expert Panel Opinion

**DOI:** 10.7759/cureus.82836

**Published:** 2025-04-23

**Authors:** Mohamad C Armouch, Ahmed M Atef, Osama Abdel Hameed, Nada A Alshaikh, Ahmed S Elbeleidy, Benkheder Ali, Lubna El Najjar, Eslam Tawfik

**Affiliations:** 1 Otolaryngology, Mediclinic Al Noor Hospital, Abu Dhabi, ARE; 2 Otolaryngology, Faculty of Medicine, Cairo University, Cairo, EGY; 3 Otolaryngology, Faculty of Medicine, Ain Shams University, Cairo, EGY; 4 Otolaryngology, Mouwasat Hospital, Dammam, SAU; 5 Pediatrics, Faculty of Medicine, Cairo University, Cairo, EGY; 6 Pulmonology, Faculty of Medicine of Tunis, Tunis, TUN; 7 Medical Affairs, Sanofi, Dubai, ARE

**Keywords:** allergic rhinitis, antihistamines, arab region, intranasal corticosteroids, treatment algorithm

## Abstract

Allergic rhinitis (AR) is a prevalent atopic condition that is frequently misdiagnosed, mistreated, or overlooked, despite its high prevalence and significant economic burden. Pharmacotherapy effectively controls symptoms in the majority of cases; however, the current local guidelines and recommendations are ambiguous regarding the initiation and cessation of combined antihistamines and intranasal corticosteroids treatment. Additionally, guidelines do not specify the duration of AR therapy or identify cases eligible for combination therapy. In contrast, the current era emphasizes personalized medicine, which considers genetic variants that may clinically alter the tolerance and intended outcome of specific drugs. Moreover, there has been a noticeable surge in the number of individuals affected by AR in the Arab region. A systematic treatment algorithm tailored to the Arab region’s population is urgently needed. This consensus outlines the recommendations of experts from three scientific meetings that gathered specialists in otolaryngology, pediatrics, and immuno-allergology from Egypt, Tunisia, the Kingdom of Saudi Arabia, and the United Arab Emirates. The expert panel opinion encompassed pre-meeting surveys followed by discussions about diversified topics related to AR management in the Arab region, including AR diagnosis and disease characteristics, patient profiling, and conventional and advanced treatment options. The experts developed a streamlined algorithm to enhance decision-making for AR treatment in Arab countries, based on discussions and a comprehensive literature review. To our knowledge, this manuscript presents the first region-specific, consensus-based management algorithm tailored to the Arab region, addressing unique local considerations.

## Introduction

Allergic rhinitis (AR) is an inflammatory disorder affecting the nasal mucosa, induced by inhaled allergens through IgE-mediated hypersensitivity responses [1]. Symptoms of AR include one or more of the following: nasal itching, rhinorrhea, sneezing, and/or nasal congestion, accompanied by other non-nasal symptoms involving the eyes, ears, and throat [2]. The pathophysiology of AR is primarily driven by prolonged exposure to threshold levels of various allergens, such as cockroach allergens, pollen, and dust mite fecal proteins. This exposure facilitates the presentation of allergens by antigen-presenting cells (APCs) to CD4+ T-lymphocytes. Once activated by APCs, these T-lymphocytes release TH2 cytokines, including IL-3, IL-4, and IL-5 [2]. The TH2 cytokines, in turn, promote proinflammatory processes against these allergens, which include the production of IgE and the rapid multiplication of neutrophils, mast cells, and eosinophils [2,3]. The produced IgE then attaches to the high-affinity IgE receptors on basophils or mast cells [3]. There is a significant genetic component to allergic sensitivity that distinguishes AR [2]. This implies that the patient’s inherited genetic makeup determines the immune response pattern to allergens [2].

Furthermore, AR stands as one of the most prevalent medical conditions globally, impacting approximately 40% of adults. Currently, it holds the position of the fifth most prevalent chronic disease in the United States overall, and it is the most prevalent chronic disease among children worldwide, affecting around 25% of them [1,4]. The prevalence of AR has been on the rise over the years, attributed to factors such as increasing pollution levels and climate change. Moreover, AR has a substantial economic impact, with a total annual cost of €1.3 billion and $20.9 billion in Sweden and the United States, respectively [1]. On the other hand, regarding the prevalence of AR in the Arab population, a self-reported prevalence of 36% was observed [5]. This notably high frequency of AR in the study may be attributed to genetic factors and the influences of modernization [5].

The management of AR encompasses patient education, allergen avoidance, pharmacotherapy, allergen immunotherapy (AIT), and acupuncture [4,6]. Pharmacotherapy is often deemed effective by most patients in controlling symptoms, and when used appropriately, it significantly enhances the quality of life (QoL) [6]. Numerous therapeutic options are available for the management of AR, including oral and/or intranasal H1-antihistamines, intranasal corticosteroids (INCS), decongestants, leukotriene receptor antagonists (LTRAs), various combinations of these treatments, AIT, and biologics [4,6]. AR patients are mostly encountered and treated by general practitioners (GPs) and non-specialized physicians, only presenting to ENT specialists in severe, challenging cases [7]. Therefore, expert ENTs collaborated in crafting a pathway that would largely unify and facilitate the AR patient journey in the region, from initial treatment until specialist referral.

## Materials and methods

**Figure 1:**
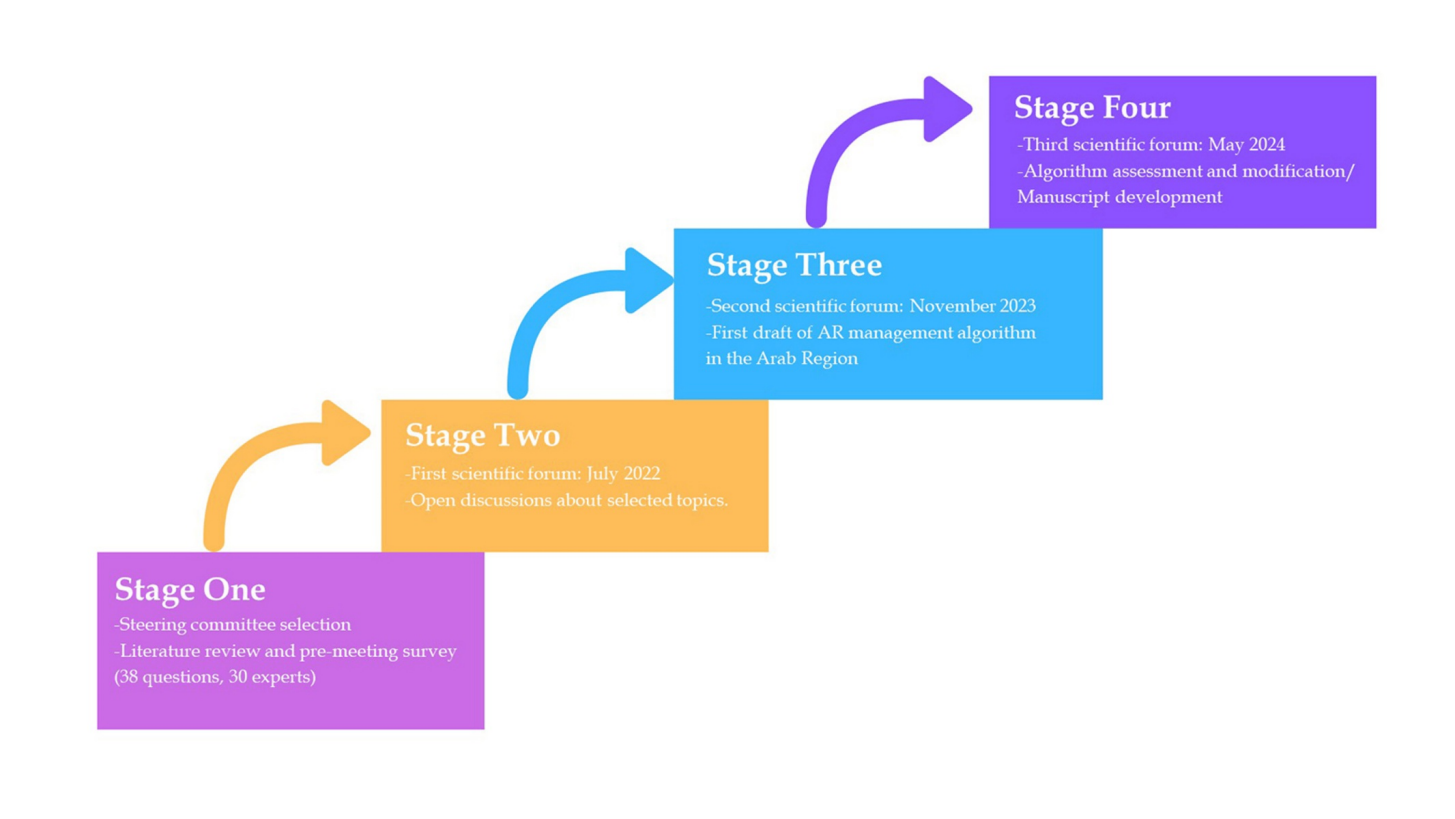
Stages of developing a framework for AR management in the Arab region AR, allergic rhinitis

The four main stages involved in the manuscript and algorithm formulation process are illustrated in Figure 1, reflecting a systematic approach to developing a robust framework for AR management. This iterative process ensures that the outcome remains clinically relevant and evidence-based, ultimately contributing to enhanced patient care.

Pre-meeting questionnaire

To enhance the effectiveness and value of scientific forums focused on AR, a pre-meeting questionnaire was developed based on a literature review, with the objective of gathering insights on potential discussion topics, identifying areas of consensus and dissent, and highlighting aspects to be included in the management algorithm. The questionnaire, consisting of 35 multiple-choice questions, was distributed via email to a panel of 30 field experts recruited through the authors’ professional network. All questions and answer submissions were anonymized to ensure unbiased and independent responses. Next, the answers were analyzed and displayed as percentages. The topics evaluated within the pre-meeting questionnaire included AR disease characteristics, the impact of AR on patients’ QoL, major factors influencing treatment decisions, treatment options and their associated side effects, and considerations regarding refractory AR and immunotherapy.

Scientific forums

This manuscript and the algorithm were formulated based on recommendations from a diverse group of experts in the fields of otolaryngology, pediatrics, and immuno-allergology from various countries in the Arab region, including Egypt, Tunisia, the Kingdom of Saudi Arabia, and the United Arab Emirates. Collaboration occurred during a series of three expert forums held in July 2022, November 2023, and May 2024.

The selection of experts for participation was based on their extensive knowledge of ENT disorders and their expertise in the management of AR. Additionally, those chosen demonstrated a commitment to the innovation of solutions aimed at improving patient QoL and clinical outcomes. During the initial two forums, the experts conducted a literature review followed by a systematic discussion regarding critical topics, including disease diagnosis, patient characteristics linked to AR, classification of AR and its impact on treatment choices, the impact of AR on patients’ QoL, various treatment modalities and combination therapies, and special considerations in the management of AR.

Development of the AR management algorithm

A preliminary draft of the AR management algorithm was initiated during the first two forums. The development process of the algorithm can be delineated into the following stages:

Identification of Key Components

Based on the literature review and discussions held, essential elements pertaining to AR management were identified. This included factors such as diagnostic criteria, optimal disease classification elements, and the most suitable treatment options for each group of patients.

Drafting the Algorithm

An initial version of the algorithm was constructed, bringing together the identified components into a cohesive framework that reflects the collective insights of the participating experts.

Evaluation and Feedback

The draft algorithm was subjected to evaluation in the subsequent forum. Expert feedback was solicited to identify gaps and areas for improvement in the initial draft.

Refinement and Finalization

During the third forum, the algorithm underwent a comprehensive refinement and finalization process, marking the fourth stage of development. This forum primarily ensured clarity, applicability, and effectiveness and encompassed implementing the algorithm into the manuscript’s draft.

## Results

Pre-meeting survey questionnaire

Results (Appendix A) revealed that all 30 participants answered 19/35 questions of the pre-meeting survey, 29 participants answered 7/35 questions, and 26 participants answered 9/25 questions. Highlights from the pre-meeting questionnaire included the agreement on the high prevalence of AR in the region, ranging from 10% to 30% of the Arab population. Additionally, the superiority of INCS over antihistamines and LTRA in controlling the symptoms of AR was noted. It was also suggested that immunotherapy can be initiated when symptoms persist for an extended period without improvement and the patient wishes to either discontinue or titrate down/reduce their doses. The consensus levels in the questionnaire regarding topics like AR classification and treatment choices highlighted specific areas of disagreement that warrant more extensive discussion in the forums.

Summary of the scientific forums

The scientific forums featured experts and included discussions on the following topics:

Diagnosis and Characteristics

Although the diagnosis of rhinitis necessitates a detailed medical history and physical examination, additional diagnostic testing is required to demonstrate that underlying allergens cause the rhinitis [8]. Skin-prick testing is the most common technique for determining certain allergy triggers of rhinitis [8]. Regarding imaging, the use of X-rays is obsolete in the diagnosis of patients with AR, even in comorbid patients. Implementing X-ray scanning is limited to pediatric cases where a stuck foreign body is suspected. Besides AR, X-rays are implemented for the differential diagnosis of adenoids [9]. On the other hand, CT scans are only used if needed.

**Figure 2:**
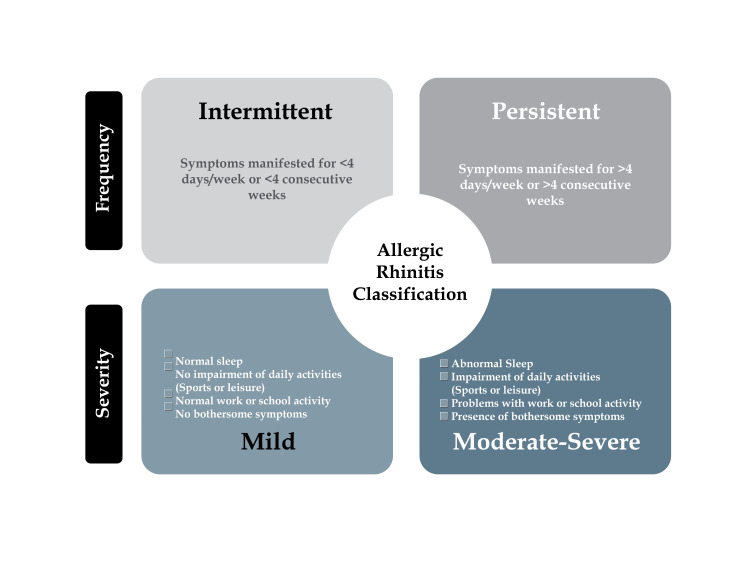
AR disease stratification based on symptom pattern and severity AR, allergic rhinitis

Disease classification: AR can be classified based on frequency into intermittent and persistent instead of seasonal and perennial to ensure familiarity, since these are the most widely used classifications. As illustrated in Figure 2, AR can be further subdivided based on severity into mild, moderate, or severe. This classification is consistent with the classification published by the Allergic Rhinitis and its Impact on Asthma (ARIA) guidelines [10]. Intermittent AR is defined by symptoms manifested for <4 days/week or <4 consecutive weeks, while persistent AR is characterized by symptoms present for >4 days/week or >4 consecutive weeks [11]. Furthermore, mild symptoms can be defined as having normal sleep, no impairment of daily activities, whether sports or leisure, normal work or school activity, and no bothersome symptoms. On the other hand, moderate to severe symptoms can be described by patients exhibiting abnormal sleep, impairment of daily activities, whether sports or leisure, problems with work or school activity, and bothersome symptoms. The disease classification was consistent with the disease classification provided by the Practical Guide for Allergy and Immunology in Canada 2018 [8] and Bousquet et al. (2008) [12]. AR patient profiles’ stratification can involve a categorization based on the disease manifestations into blockers, who predominantly exhibit nasal congestion, and runners, who predominantly demonstrate sneezing and rhinorrhea [13,14]. This classification improves the treatment decision between antihistamines and nasal decongestion.

Disease burden and impact on QoL: Many AR patients have an impaired QoL since the disease significantly impacts their sleep quality and cognitive function and causes irritability and fatigue [15,16]. Furthermore, AR is associated with decreased school and work performance, especially during the peak of the pollen season [15].

AR treatment modalities: conventional pharmacotherapies

The conventional therapeutic modalities employed in the management of AR typically involve H1-antihistamines, INCS, decongestants, and combination therapies [17]. Firstly, second-generation oral antihistamines (e.g., fexofenadine, desloratadine, cetirizine, and loratadine) are recommended over first-generation antihistamines (e.g., chlorpheniramine and diphenhydramine) owing to the latter’s negative impact on cognition [4,8]. Therefore, second-generation oral antihistamines were recommended as the first-line pharmacological treatment for all patients with mild intermittent AR [4,8]. Intranasal antihistamines, namely azelastine and olopatadine, offer the advantage of delivering a higher drug concentration to a targeted area than oral antihistamines, which translates into fewer side effects and a faster onset of action within 15 minutes. Therefore, in moderate/severe AR cases, intranasal antihistamines are beneficial in instances where rapid symptom relief is required [4,17].

It is important to consider the approval status of using intranasal antihistamines in local guidelines before using intranasal antihistamines in pediatrics. For instance, the FDA approves the use of azelastine for patients aged five and older and olopatadine for patients aged six and older [17]. Moreover, INCS has been shown to be the most effective category of medications for alleviating the symptoms of AR [18,19]. INCS is regarded as a primary treatment choice for persistent AR of any severity or moderate to severe intermittent AR [4,20]. In contrast, LTRAs, such as montelukast and zafirlukast, should not be offered as a first-line treatment for AR patients. However, they have been approved for patients concurrently diagnosed with asthma [14]. INCS are generally more efficient than LTRAs and H1-antihistamines [6]. INCS do not show any systemic adverse effects since they are not systemically absorbed [6].

The most frequent localized side effects of INCS, including nasal stinging, irritation, or epistaxis, might be typically avoided by pointing the spray slightly away from the nasal septum [6,21]. Additionally, switching to a water-based spray rather than an alcohol-based spray can minimize local side effects [22]. Intranasal decongestants (oxymetazoline and phenylephrine) and the combination of oral H1-antihistamines (cetirizine hydrochloride, acrivastine, or desloratadine) with oral decongestants (pseudoephedrine) can be used when nasal decongestion is a dominant symptom [4,6,23]. Oral decongestants are not recommended for pediatrics and patients with high systemic blood pressure, prostate diseases, or cardiac conditions [24]. Oral decongestants should not be administered for more than one week [24].

AR advanced treatment options

AIT is an advanced treatment modality that potentially provides long-term relief from AR [25]. AIT encompasses two main types: subcutaneous immunotherapy (SCIT) and sublingual immunotherapy (SLIT) [25]. Immunotherapy is usually employed for intractable AR following conventional pharmacological options. SCIT and SLIT are usually safe, although local reactions are commonly reported regardless of the delivery mode. Serious, life-threatening reactions related to immunotherapy are rare [25,26]. The setting for administering immunotherapy depends on its form of administration, where SCIT is administered at the hospital, whereas SLIT could be administered at home after providing thorough patient education [25].

Management of AR in pregnancy and special considerations

Pregnancy increases fluids in the female’s body, which aggravates nose blockage, resulting in more suffering when compared with that of non-pregnant patients [27]. In the management of pregnant or breastfeeding women, some clinicians contact gynecologists to determine the suitable dose and type of prescribed antihistamines. Pregnant females are usually concerned with administering medications during their pregnancy due to the risk of teratogenicity, and hence, they are reassured by their gynecologists’ approval of the administered AR medications. Some second-generation antihistamines are considered safe to be administered during pregnancy and are categorized as category B in the treatment guidelines [28,29]. Moreover, because of its proven safety record, budesonide is typically chosen over alternative ICS for pregnant women [30].

Furthermore, age is an important factor in the treatment decision-making process. For instance, the administration of first-generation antihistamines to children should be avoided due to their sedating effect [31]. Adjunct therapies, such as topical saline or nasal aspirators, should be considered in managing AR children [32]. First-generation antihistamines should not be prescribed for working adults, especially pilots and individuals who operate vehicles or machinery [33]. Hepatic and renal functions should be assessed in elderly patients before medication prescriptions.

Recommendations for AR management: algorithm formulation

**Figure 3:**
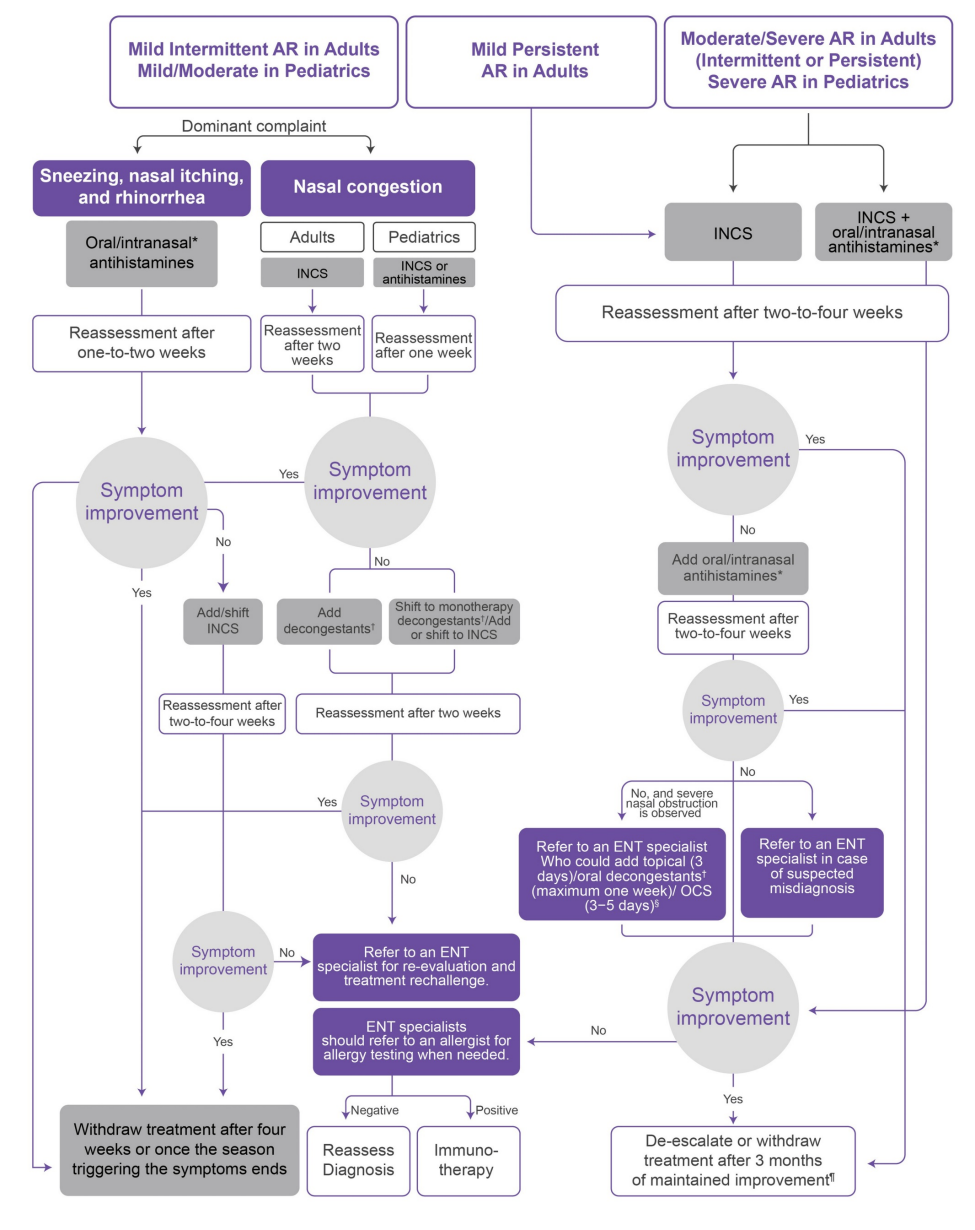
AR management algorithm in the Arab region ^*^ Intranasal antihistamines should not be used in pediatrics if not approved within the local guidelines; in moderate/severe AR cases, intranasal antihistamines are beneficial in instances where rapid action is required. ^†^ Oral decongestants are only prescribed if not contraindicated and the patient’s health condition is suitable. ^§^ OCSs (prednisolone) can be given at lower doses (0.5-40 mg/kg) for three to five days in AR patients compared with other diseases. ^¶^ Some ENTs recommend treatment de-escalation followed by dose maintenance, while others recommend treatment de-escalation followed by withdrawal and readministering the treatment when symptoms recur. AR, allergic rhinitis; OCS, oral corticosteroids

A simplified management algorithm was developed based on the experts’ recommendations and revisions during the three waves of scientific meetings. The algorithm (Figure 3) merges the management of adult with pediatric AR patients. However, it should be noted that pediatric patients are classified into two arms (mild/moderate and severe AR) based on the impact of the symptoms on the child’s activity and sleep, whereas adults are classified into mild intermittent, mild persistent, and moderate/severe intermittent and persistent AR.

Mild/intermittent AR in adults and mild/moderate in pediatrics

Sneezing, Nasal Itching, and Rhinorrhea as the Dominant Complaints

In cases of mild/intermittent disease in adults or mild/moderate disease in pediatrics, where sneezing, nasal itching, and rhinorrhea are the dominant symptoms, the administration of oral antihistamines or intranasal antihistamines is recommended. This recommendation was similar to the recommendations provided by the American Academy of Otolaryngology-Head and Neck Surgery (AAO-HNS) [4] and the Joint Task Force on Practice Parameters [9]. Reassessment is performed after one to two weeks. If no improvement is observed, INCS will be shifted to/added, and patients’ reassessment will take place after two to four weeks. The treatment options complied with the ARIA guidelines [34] and with the Japanese Society of Allergology (JSA) guidelines [35]. In case of symptom improvement, treatment can be withdrawn after four weeks or once the season triggering the symptoms ends. In contrast, if no improvement is observed afterward, the patient can be referred to an ENT specialist for reevaluation and treatment rechallenge. Finally, the ENT specialist should refer to an allergist to request an allergy test and reconsider the original diagnosis if the test result is negative or consider desensitization immunotherapy if the result is positive, which is similar to AAO-HNS guidelines [4].

Nasal Congestion as the Dominant Complaint (Post-nasal Discharge in Pediatrics)

Adults: Treatment is initiated with INCS, and patients should be reassessed after two weeks of treatment. If no improvement is observed after two weeks, a decongestant could be added to the treatment regimen. An allergy test would be requested if no improvement was observed after two weeks. The physician should reconsider the original diagnosis if the test result is negative or consider desensitization immunotherapy if the result is positive.

Pediatrics: Treatment is initiated with monotherapy antihistamines or INCS. Patients should be reassessed after one week of treatment. If no improvement is observed, patients initially treated with monotherapy antihistamines should be added to or shifted to INCS. In cases where an antihistamine was primarily initiated, treatment should encompass or be shifted to an INCS or shifted to decongestant monotherapy if INCS or antihistamines were initially initiated. Patients should be reassessed after two weeks of treatment. If no improvement is observed after two weeks, the physician should request an allergy test. The physician should reconsider the original diagnosis if the test result is negative or consider desensitization immunotherapy if the result is positive.

Moderate/severe AR in adults (only severe in pediatrics) or mild persistent AR

In a similar way to AAO-HNS [4], ARIA [34], and JSA [35], treatment is initiated with INCS. Patients should be reassessed after two to four weeks of treatment, approximating the JSA reassessment time (two to four weeks) [35]. If no improvement is observed after this period, oral or intranasal antihistamines could be added to the treatment regimen. Patients should be reassessed after two to four weeks of treatment. If no improvement is observed after two to four weeks and there is severe nasal obstruction, the patient should be referred to an ENT specialist who might add topical (three days)/oral decongestants for a maximum of one week or oral steroids for three to five days. Oral decongestants are only prescribed if they are not contraindicated and the patient’s health condition is suitable. An allergy test would be requested if no improvement was observed after two to four weeks. The physician should reconsider the original diagnosis if the test result is negative or consider desensitization immunotherapy if the result is positive.

Available guideline comparison

To critically assess the algorithm created according to the experts’ recommendations for treating AR, the algorithm was compared to an established guideline presented by AAO-HNS [4], as shown in Table 1.

**Table 1:** Comparison of the formulated algorithm with the AAO-HNS algorithm AAO-HNS, American Academy of Otolaryngology-Head and Neck Surgery; AR, allergic rhinitis; INCS, intranasal corticosteroids

Mild AR													Moderate/severe AR							
Algorithm	Main complaint is nasal itching and rhinorrhea					Main complaint is nasal congestion														
	First-line treatment	Reassessment time	Stepping up therapy (if symptoms persist)	Second reassessment time	Decision in therapy failure	First-line treatment	Reassessment time	Stepping up therapy (if symptoms persist)	Second reassessment time	Decision in therapy failure	Do pediatrics differ from the adult population?	First-line treatment		Reassessment time	Stepping up therapy (if symptoms persist)	Second reassessment time	Second stepping up therapy in case of symptom persistence	Third reassessment time	Decision in therapy failure	Do pediatrics differ from the adult population?
AAO-HNS [4]	Oral or intranasal antihistamines [4]	NA	INCS alone [4]	NA	Consider allergy testing and immunotherapy [4]	INCS [4]	NA	Add an oral decongestant (no longer than three days) [4]	NA	Consider allergy testing and immunotherapy and reassess for anatomic nasal obstruction or non-allergic inflammation [4]	The guideline can be applied to both pediatric (above two years) and adult populations [4].	INCS [4]		NA	Add oral or intranasal antihistamines [4]	NA	NA	NA	Consider allergy testing and immunotherapy [4]	The guideline can be applied to both pediatric (above two years) and adult populations [4].
Experts’ recommendations - Arab region (adults)	Oral or intranasal antihistamines	Two to four weeks	Add or shift INCS	Two to four weeks	Refer to an ENT specialist for (1) reevaluation and treatment rechallenge and (2) referral to an allergist for allergy testing and possible immunotherapy	INCS	Two weeks	Add decongestants	Two weeks	Refer to an ENT specialist for (1) reevaluation and treatment rechallenge and (2) referral to an allergist for allergy testing and possible immunotherapy	The management in the pediatric population differs from the adult population.	INCS		Two to four weeks	Add oral or intranasal antihistamines	Two to four weeks	In case of severe nasal obstruction, add topical (three days) or oral decongestants (one week) or oral steroids (three to five days).	Two to four weeks	Consider allergy testing and immunotherapy	This management strategy is applied only in moderate to severe cases in adults and applied in severe cases in pediatrics.

In the beginning, Table 1 demonstrated that experts’ recommendations provided clear AR management in pediatrics; this was not the case in the AAO-HNS’ guidelines, which presumed the applicability of adults’ management steps in pediatrics. Moreover, the experts’ recommendations provided definite intervals to reassess therapy effectiveness, while the other guidelines did not provide it. However, the two algorithms similarly classified the patient’s symptom frequency into mild and moderate-to-severe symptoms. The reason why they did not classify AR symptoms into intermittent (<4 days per week or <4 weeks per year) and persistent (>4 days per week and >4 weeks per year) was explained in the AAO-HNS guidelines [4], which illustrated the limitations of this classification. For example, a patient may be classified as intermittent despite displaying symptoms for three days per week, year-round. This patient profile would be closer to a persistent patient rather than an intermittent [4]. Furthermore, first-line therapies were identical in both guidelines for mild (oral or intranasal antihistamines) and moderate/severe (INCS) AR, in addition to the decisions made in case of therapy failure (allergy testing and immunotherapy).

## Discussion

Despite the presence of global guidelines for the proper management of AR, no region-specific guidelines exist for the Middle East [36]. Furthermore, there is a need for an evidence-based management approach and greater community knowledge of the disease. Therefore, this algorithm assists GPs and non-specialized physicians in managing AR patients, helping to standardize clinical practice across the region. The experts created a treatment algorithm for adults and pediatrics above two years of age. This management model consolidates the therapeutic options used for both adults and children in certain areas while also recognizing the differences in other aspects. First of all, in adults, AR was classified into mild/intermittent AR and moderate/severe AR. On the other hand, in pediatrics, AR was classified into mild/moderate and severe. In the case of mild/intermittent AR in adults or mild-moderate in pediatrics, a further subclassification was performed according to the dominant complaint. These two subtypes included predominantly patients who were runners who often reported sneezing, nasal itching, and rhinorrhea as the main complaints, and patients who were predominantly blockers who were troubled by nasal congestion as the main complaint.

The primary therapeutic options varied across the different classes of AR. This is exemplified in adult patients with mild/intermittent AR and pediatric patients with mild-moderate AR, both having sneezing and nasal itching as dominant complaints; oral or intranasal antihistamines were decided to be the first-line therapeutic approach. In addition, in adult patients with mild/intermittent AR and nasal congestion as the main complaint, INCS was set as the first-line treatment. Moreover, in pediatric patients with mild to moderate AR and nasal congestion as the main complaint, first-line monotherapy was either INCS or decongestants. Furthermore, INCS was determined to be the first-line treatment option in adults with moderate/severe AR and in pediatrics with severe AR.

The reassessment period was scheduled to occur after each step of the therapy journey. The minimum interval for reassessment was one week, while the maximum could extend up to four weeks. In the case of refractory AR, the two options advised were either immunotherapy or reconsidering the diagnosis. This treatment consensus outlines a simplified approach aimed at offering patients in the Arab region more accessible care that aligns with their needs and effectively alleviates their symptoms.

## Conclusions

Creating a localized treatment algorithm is essential for aiding physicians in managing various AR conditions. This necessity has become increasingly urgent due to the substantial rise in AR cases, the unique genetic profiles of the Arab region’s population, and the lack of local guidelines. Therefore, this consensus provides the first region-specific AR management algorithm tailored to the Arab population, addressing key limitations in existing international guidelines. By incorporating expert consensus and region-specific clinical factors, this framework has the potential to standardize AR management, improve patient outcomes, and enhance physician decision-making in the region.

## Appendices

Appendix A

**Table 2:** Pre-meeting questionnaire results AH, antihistamines; AR, allergic rhinitis; ARIA, Allergic Rhinitis and its Impact on Asthma; ICS, inhaled corticosteroids; INAH, intranasal antihistamines; INCS, intranasal corticosteroids; LTRA, leukotriene receptor antagonist; QoL, quality of life; RAST, radioallergosorbent test

Aspect covered	Question	Options	Results
Disease characteristics	AR is the most common form of rhinitis and is expected to affect ~10-30% of the population (adults and pediatrics).	Agree	100%
		Disagree	Null
	AR is a chronic disease that could be controlled but not cured.	Agree	80%
		Disagree	20%
Disease diagnosis	AR diagnosis is mainly clinical (physical examination is important).	Agree	89.70%
		Disagree	10.30%
	Is an X-ray in the diagnosis of AR mandatory?	Agree	6.70%
		Do not agree	55.30%
		Needed sometimes	40%
Disease classification	Which is the most recent/accepted classification for AR?	Frequency (intermittent or persistent) and severity	62%
		IgE-mediated (allergic), autonomic, infectious, and idiopathic (unknown)	24%
		Seasonal (occurs during a specific season) or perennial (occurs throughout the year)	14%
	“Intermittent” AR as symptoms that are present:	Less than four days per week or for less than four consecutive weeks	63.30%
		Less than four days per month	23.30%
		Less than four weeks per year	6.70%
		None of the above	6.70%
	“Persistent” AR as symptoms that are present:	Less than 4 days per week or for less than 4 consecutive weeks	33.30%
		Less than four weeks per year	6.70%
		Less than 4 days per month	3.30%
		None of the above	56.70%
	Mild AR as symptoms that are present:	No impairment of daily activities, sports, and leisure	13.30%
		Normal work/school	10%
		Normal sleep	3.30%
		No nasal blockage, no postnasal discharge, no throat itching, and no eye symptoms	Null
		No bothersome symptoms	Null
		All of the above	73.40%
	Moderate AR as symptoms that are present:	Nasal blockage, postnasal discharge, throat itching, and eye symptoms are the main symptoms	51.70%
		No bothersome symptoms	7%
		No impairment of daily activities, sports, and leisure	3.40%
		Normal work/school	3.40%
		No nasal blockage, no postnasal discharge, no throat itching, and no eye symptoms	Null
		Normal sleep	Null
		All of the above	34.50%
	AR reduces the QoL of many patients, impairing the following:	Sleep quality and cognitive function and cause irritability and fatigue. AR is associated with decreased school and work performance, especially during the peak pollen season.	36.70%
		Annual direct medical costs of AR are substantial, but indirect costs associated with lost work productivity are greater than those incurred by asthma.	3.30%
		AR is a frequent reason for physicians’ general practice office visits.	Null
		All of the above	60%
	The choice of treatment would mostly depend on:	Proven efficacy, patient preference, local availability, and cost of treatment	80.80%
		Updated options	15.40%
		Cheaper options	3.80%
	In patients with perennial (persistent) AR/severe intermittent, ARIA suggests either a combination of an INCS with an intranasal H1 AH or an INCS alone; the determinant factor is:	Proven efficacy, patient preferences, local availability, and cost of treatment	61.50%
		Brand type	7.70%
		Physician’s preference	7.70%
		All of the above	23.10%
	As regards seasonal “mild/moderate intermittent” AR, at the initiation of treatment (~first two weeks), AH is preferred to start with or to be included:	The inclusion of AH at the initiation of seasonal mild/moderate intermittent AR treatment reduces symptoms and improves QoL.	27%
		Combining AH with ICS acts faster than ICS alone.	15.40%
		Both of the mentioned options	53.80%
		Fewer side effects	3.80%
		They are cheaper.	Null
		They can be more convenient.	Null
	In patients with seasonal “mild/moderate Intermittent” AR, ARIA suggests that if the decision of using either an LTRA or an AH, LTRA might be preferred only in one condition	LTRA is preferred over AH only in patients who have concomitant asthma, especially exercise-induced and/or aspirin-exacerbated respiratory disease.	80.80%
		Cardiac patients	7.70%
		Patients living in the Northern Hemisphere	7.70%
		Athletes	3.80%
Mild/intermittent AR	In patients with seasonal “mild/moderate intermittent” AR, ARIA suggests starting with an oral AH or combination of an INCS with an oral AH.	Agree	70%
		Disagree	30%
	Assessment of mild intermittent AR treatment with oral AH should not exceed one to two weeks.	Agree	73%
		Disagree	27%
	The first-line pharmacological treatments recommended for all patients with mild/intermittent AR	Second-generation oral antihistaminic	40%
		Allergen avoidance	23.30%
		INCS	23.30%
		INAH	13.40%
		Oral corticosteroids	null
Perennial (persistent)/severe intermittent AR	In patients with perennial (Persistent) AR/severe intermittent, ARIA suggests starting with an INCS alone rather than a combination of an INCS with an AH (conditional recommendation | very low certainty of evidence).	Agree	58%
		Disagree	42%
	In patients with perennial (persistent)/severe intermittent AR, ARIA suggests an oral H1-AH rather than an LTRA (except in asthmatic patient).	Agree	77%
		Disagree	23%
Oral AH	Although the older (first-generation) AH (e.g., diphenhydramine and chlorpheniramine) are also effective in relieving symptoms, they are not recommended for the treatment of AR because:	They have anti-cholinergic and extrapyramidal side effects	70%
		They have no effect on symptoms.	6.70%
		They are old and expected to have expired.	3.30%
		None of the aforementioned reasons were relevant.	20%
INAH	INAH drugs could be used in pediatrics of age above six years.	Agree	88%
		Disagree	12%
LTRA	Regarding LTRAs, montelukast and zafirlukast can be prescribed in the treatment of AR; however;	LTRAs (montelukast and zafirlukast ) do not appear to be as effective as INCS and should not replace them.	63.30%
		They are more effective than INCS.	6.70%
		None of the above	26.70%
		Should be used as first-line therapy	3.30%
	LTRAs are best indicated when oral AH and INCS (monotherapy or combined) are there with underlying Asthma symptoms	Agree	100%
		Disagree	Null
INCS	INCS could be considered also as a first-line therapeutic option in the following:	All types of AR	50%
		INCS can be used in persistent AR (any severity) or moderate/severe intermittent AR.	43.30%
		INCS should never be used as a first line.	6.70%
		Mild intermittent AR	Null
	Studies and meta-analyses have shown that, when used regularly and correctly, INCS	INCS are superior to AH and LTRA in controlling the symptoms of AR, including nasal congestion and rhinorrhea.	83.30%
		INCS are not superior over intranasal AH.	6.70%
		INCS are not superior over LTRA.	Null
		None of the above	10%
	The most common side effects of INCS are:	Nasal dryness and epistaxis are the most common side effects	66.70%
		Dependence	16.60%
		Severe rebound of symptoms	10%
		Systemic complications such as Cushing syndrome	6.70%
	INCS side effects can usually be prevented by:	Aiming the spray slightly away from the nasal septum	79.30%
		Oral corticosteroids	10.30%
		Not using INCS	7%
		Replacing INCS	3.40%
AR symptomatic treatment	Symptomatic treatment for AR (nasal decongestant, nasal drops, and sprays) should not be prescribed for more than five days to avoid any possible complications.	Agree	83.30%
		Disagree	16.70%
	What is the last medical resort for refractory AR?	Immunotherapy desensitization	76.70%
		AH	10%
		Oral steroids	10%
		INCS	3.30%
	In your opinion, immunotherapy stops the symptoms completely.	Agree	59%
		Disagree	41%
	Where should immunotherapy be conducted?	Subject to the type of immunotherapy	63.30%
		At hospital	23.40%
		At home	13.30%
	Immunotherapy is very risky and affects the immunity of patient.	Agree	53.30%
		Disagree	46.70%
	What is your approach when choosing immunotherapy for your eligible patients?	Preparing the extract in their clinics	30%
		Choosing the most expensive immunotherapy available	10%
		None of the above	60%
	From your practice, when can you start immunotherapy?	When symptoms cannot be improved or last for a long time, and the patient wants either to stop or alleviate the medication	93.40%
		Directly after the first failure	3.30%
		Every case of AR	3.30%
	Prick (skin) and/or RAST are the main key tests before starting the immunotherapy.	Agree	86%
		Disagree	14%
